# Comparative analysis of endogenous hormones level in two soybean (*Glycine max* L.) lines differing in waterlogging tolerance

**DOI:** 10.3389/fpls.2015.00714

**Published:** 2015-09-17

**Authors:** Yoon-Ha Kim, Sun-Joo Hwang, Muhammad Waqas, Abdul L. Khan, Joon-Hee Lee, Jeong-Dong Lee, Henry T. Nguyen, In-Jung Lee

**Affiliations:** ^1^Division of Plant Biosciences, Kyungpook National UniversityDaegu, South Korea; ^2^Division of Plant Sciences and National Center for Soybean Biotechnology, University of MissouriColumbia, MO, USA; ^3^Department of Agriculture, Government of Khyber PakhtunkhwaPakistan; ^4^UoN Chair of Oman's Medicinal Plants and Marine Natural Products, University of NizwaNizwa, Oman

**Keywords:** abscisic acid, aerenchyma cell, adventitious root, ethylene, gibberellin

## Abstract

Waterlogged condition due to flooding is one of the major abiotic stresses that drastically affect the soybean growth and yield around the world. As a result, many breeders have focused on the development of waterlogging tolerance in soybean varieties, and thus, several tolerant varieties were developed. However, the physiological mechanism of waterlogging tolerance is not yet fully understood. We particularly studied the endogenous hormones regulation during waterlogging in two contrasting soybean genotypes. According to our results, adventitious roots were better developed in the waterlogging tolerant line (WTL) than in the waterlogging susceptible line (WSL). Endogenous hormones also showed significant differences between WTL and WSL. The ethylene production ratio was higher in WTL than in WSL, and methionine was higher in WTL than in WSL. Other endogenous abscisic acid (ABA) contents were lower in WTL than in WSL. Conversely, gibberellic acid (GA) showed a tendency to be high in WTL, especially the levels of the bioactive GA_4_. The ratio of total GA and ABA was significantly higher in WTL than in WSL. Anatomical study of the root revealed that aerenchyma cells in the stele were better developed in WTL than in WSL.

## Introduction

Soybean is one of the most important edible oil-producing leguminous crops that gained tremendous applause due to its nutritional values (Waqas et al., 2014). Soybean contains protein, oil, insoluble carbohydrate, soluble carbohydrate, moisture, ash as well as various functional materials such as anthocyanins, isoflavones, saponin, and dietary fiber (Thomas et al., 2003; Bellaloui et al., 2013; Waqas et al., 2014). Particularly, soybean seeds comprise high amount of protein, and they are one of the main sources of protein to humans. All these factors made soybean one of the dominant crops and is, therefore, cultivated worldwide (Bellaloui et al., 2013).

For obtaining maximum yeid, various cultivation factors including soil condition, irrigation, fertilizer supply, protection from fungal and insect attack, environmental condition etc., have to be optimized (Chen et al., 2011). Cultivation factors such as soil condition, irrigation, fertilizers supplementation, and crop protection from disease can be modulated through human efforts; however, controlling the environmental conditions in a cultivation area is almost impossible (Chen et al., 2011; Fan et al., 2012; Ahmed et al., 2013). Environmental conditions in cultivation areas are rapidly degrading and mainly attributed to the global climate change. As a result, various meteorological disasters including serious drought, salinity, unusual high and low temperatures, and freezing and flooding events constantly occur in crop cultivation areas (Fan et al., 2012). To overcome these poor environmental conditions, agriculture scientists are constantly trying to develop new crop varieties that can adapt to or are tolerant to unfavorable environmental conditions (Ahmed et al., 2013).

Flood stress, especially, is a primary restricting factor in soybean cultivation because 60–70% of the annual precipitation occurs in the summer season, the period of soybean growth and development. According to Nguyen et al. (2012), waterlogging stress during vegetative stage of soybean growth causes a reduction in grain yield of approximately 17–40% and that during the reproductive stage led to a 40–57% yield reduction.

In cultivation area, flooding stress is caused by two different events: (1) submergence stress, where the plant organ is completely under water, and (2) waterlogging stress, where the plant leaf and stem are partially submerged under water (Nishiuchi et al., 2012). Under the first stress condition, the plant experiences poor environmental conditions, such as the limitation of gas exchange, low light intensity, increased susceptibility to fungal diseases, and the disturbance of nutrients uptake from the soil (Ram et al., 1999; Nishiuchi et al., 2012). This kind of submergence event normally occurs in subaquatic plants. On the other hand, waterlogging stress frequently occurs during general crop cultivation due to various external environmental factors such as heavy rain fall, excess irrigation, storms, and overflow of rivers. In soybean cultivation, waterlogging stress is emerging as a primary abiotic stress, and thus, current research focuses on waterlogging stress in soybean plants. According to Alam et al. (2010), occurrence of waterlogging stress is defined as when the soil pores are saturated with water such that it becomes over soil capacity by at least 20%. Under normal growth conditions, plant roots take up oxygen from soil and then use in mitochondrial respiration. However, under waterlogging stress conditions, plants cannot absorb ample oxygen to maintain normal physiological functions. Therefore, plants cannot generate glucose and ultimately experience various metabolic problems.

In order to adapt to hypoxia, plants try to morphologically change the intercellular formation via various physiological responses. Among those physiological responses, development of aerenchyma cells in plant roots is the most typical and is induced by several signal regulators such as nitric oxide (NO), reactive oxygen species (ROS), and plant hormones (Dat et al., 2004). Particularly, according to Kreuzwieser and Rennenberg (2014), endogenous ethylene production is closely correlated with the development of aerenchyma cells. The plant hormone ethylene is biosynthesized from methionine, and it is produced by the activation of two enzymes such as 1-aminocyclopropane-1-carboxylic acid (ACC) synthase and ACC oxidase (Arc et al., 2013). In the final step of the ethylene biosynthesis pathway, the ACC, which is an intermediate product during ethylene biosynthesis, is converted to ethylene by oxidation, and therefore, oxygen is the main component for the production of ethylene. Thus, conditions of anoxia or hypoxia in plants due to flooding stress restricts ethylene production in plant roots; however, when plants are faced with stress, ethylene production is increased due to enhancement of ACC synthase (Dat et al., 2004). Increased ethylene crosstalks with other hormones such as abscisic acid (ABA), gibberellic acid (GA), auxin (IAA), and cytokinin (CK) to adapt flooding stress condition, especially anoxia or hypoxia condition, and aerenchyma cells are developed by these kind of interactions (Dat et al., 2004; Xu et al., 2006; Bailey-Serres and Voesenek, 2008; Fukao and Bailey-Serres, 2008; Shimamura et al., 2014).

As mentioned above, the physiological and morphological changes of flooding stress have been identified by various researchers; however, most of these studies are limited to subaquatic plants (rice plant) and focused on the submergence condition. Soybean and other dryland crop species also need investigation about flood stress response, especially waterlogging stress, as these are the main food consumption crops for humans and the farmlands dedicated to these crops are faced with waterlogging stress and assumed to be further deteriorated by worldwide climate change. Recent studies have identified and categorized aerenchyma cells into primary and secondary types and their functions with respect to two diverse habitat subaquatic crop like and dryland crop like soybean. Both types of primary aerenchyma, i.e., schizogenous and lysigenous formations, are promoted in subaquatic rice plant, maize, barley, and wheat roots under waterlogged condition. Contrarily, in dryland crop plants like soyabean, the secondary type aerenchyma is found and differentiated from the secondary meristem, and, the presence is observed in stem, taproot, hypocotyl, adventitious roots, and root nodules (Jackson and Armstrong, 1999; Shimamura et al., 2003, 2014; Yamauchi et al., 2013). In addition, the secondary aerenchyma enhances the formation of hypertrophic lenticels on roots (adventitious) and stem surface to provide greater surface area for entry of oxygen and thus directly increase the importance of adventitious roots formation in soybean (Yamauchi et al., 2013). The objective of this study was to conduct a comparative analysis of hormones response to waterlogging between two contrasting genotypes, with the hope that this information will pave the way for further mechanistic investigation of the role of specific hormones in waterlogging tolerance.

## Materials and methods

### Tolerance and resistance population development

Two parent lines (PI408105A and S99-2281) previously used for the development of recombinant inbred lines (RIL) tolerant to flooding and resistant to *Phytopthora sojae* Kaufm. and Gerd. (Nguyen et al., 2012) were selected to understand the hormonal interactions for waterlogging tolerance. For waterlogging stress experiments, the PI408105A, an exotic accession with tolerance to flooding, was used as a waterlogging tolerant line (WTL) and S99-2281, an elite line sensitive to flooding, was used as a waterlogging susceptible line (WSL). Shannon et al. (2005) tested more than 300 soybean germplasms to select waterlogging tolerance soybean varieties, and as a result, they identified the waterlogging tolerance line (PI408105A), which showed approximately 30% reduction of grain yield under waterlogging stress compared to 81% reduction of yield in the waterlogging susceptible line (S99-2281). For these reason, we used these two soybean lines in our study.

### Cultivation methods, growth condition, and flooding stress treatment

For the waterlogging experiments, seeds were thoroughly washed with sterilized double distilled water, and each soybean line was sown into horticultural soil (fungal-free biosoil; Dongbu Farm Hannong, South Korea) contained in plastic pots (9.5 × 9.5 × 8.5 cm). During the experimental period, soybean plants were grown in a growth chamber [temperature 32/25°C (day/night); relative humidity 65%; day-length 14/10 h (day/night); light intensity 1000 μmol m^−2^s^−1^]. Flooding stress was applied to soybean plants in the vegetative 3 (V3) stage and then the plant samples were harvested at 5th and 10th day after waterlogging stress treatment. For flooding stress application, we maintained the water level more than 5 cm above the soil surface, and for control plants, soil humidity were maintained according to established soybean cultivation methods (soybean cultivation method, Rural Development Administration, South www.rda.go.kr; Rural Development Administration, South Korea).

### Plant growth characteristics data

Shoot length, shoot fresh weight, number of adventitious roots, and stem width were used as growth characteristics data. Data were collected at 5- and 10-days intervals after waterlogging treatment. The data on shoot length and fresh weight were recorded on 20 plants per replicate, and the experiment comprised of three replications per treatment.

### Endogenous hormones analysis

#### Endogenous ethylene analysis

Fresh whole plant samples from each treatment were used for ethylene production. After 5th and 10th day of waterlogging treatment, soybean plant samples intended for ethylene analysis were carefully removed from the pots, and the attached soil was thoroughly washed with distilled water. The soil-free plant samples were then kept in conical tubes for 30 min, and gas samples were withdrawn from headspace with a plastic syringe. The extracted 1 mL gas samples were injected into GC with flame ionization detector (GC-17A, Shimadzu, Japan), and ethylene was determined by GC programed (Table 1) as mentioned in Kim et al. (2011). Caution was taken to accurately measure the ethylene evolution and not the ethanol. Therefore, before starting endogenous ethylene analysis in each sample, the GC was calibrated every time with standard ethylene to determine the exact retention time. Furthermore, for better separation of ethylene from other gas contents, a 15 m × 0.25 mm i.d. fused silica capillary column with a chemically bonded 0.25 μm DB-5-MS stationary phase (J&W Scientific, Folsom, CA, USA) was used.

**Table 1:** **GC conditions for ethylene production**.

**GC conditions**	
Equipment	GC-17A, Shimadzu, Japan; FID detector
Column	Capillary column (Rtx-1 30 m × 0.53 mm × 0.5 μm, Restek, USA)
Carrier gas	N (25 mL·min^−1^)
Injector temperature	130°C
Detector temperature	120°C
Oven temperature	110°C

#### Endogenous abscisic acid (ABA) analysis

After harvesting, the whole soybean plant samples were immediately frozen in liquid nitrogen and freeze dried (ISE Bondiro freeze dryer, Ilsin Bio Base, Yangju, South Korea). The whole plants freeze-dried samples were grinded and used for ABA, GA, SA, and JA analysis. The endogenous ABA content was analyzed according to the method of Qi et al. (1998) and Kamboj et al. (1999). The powdered whole plant samples were treated with 30 ml of extraction solution containing 95% isopropanol, 5% glacial acetic acid, and 20 ng of [(±)–3,5,5,7,7,7–d^6^]–ABA. The filtrate was concentrated by a rotary evaporator. The residue was dissolved in 4 ml of 1N sodium hydroxide solution, and then washed three times with 3 ml of methylene chloride to remove lipophilic materials. The aqueous phase, brought to approximately pH 3.5 with 6N hydrochloric acid, was partitioned three times into ethyl acetate (EtOAc). EtOAc extracts were then combined and evaporated. The dried residue was dissolved in phosphate buffer (pH 8.0) and then run through a polyvinylpolypyrrolidone (PVPP) column. The phosphate buffer was adjusted to pH 3.5 with 6N HCl and partitioned three times into EtOAc. EtOAc extracts were combined again and evaporated. The residue was dissolved in dichloromethane (CH_2_Cl_2_) and passed through a silica cartridge (Sep-Pak; Water Associates, Milford, Massachusetts, USA) pre-washed with 10 ml of diethyl ether:methanol (3:2, v/v) and 10 ml of dichloromethane. ABA was recovered from the cartridge by elution with 10 ml of diethyl ether (CH_3_–CH_2_)_2_O: methanol (MeOH) (3:2, v/v). The extracts were dried and methylated by adding diazomethane for GC/MS-SIM (6890N network GC system, and the 5973 network mass-selective detector; Agilent Technologies, Palo Alto, CA, USA) analysis (Table 2). For quantification, the Lab-Base (ThermoQuset, Manchester, UK) data system software was used to monitor responses to ions of *m*/*e* 162 and 190 for Me-ABA and 166 and 194 for Me-[^2^H_6_]-ABA.

**Table 2:** **GC-MS-SIM conditions for the determination of endogenous ABA, GA, and JA**.

**GC-MS-SIM conditions**	
Equipment	Hewlett-Packard 6890, 5973N Mass Selective Detector
Column	DB-1 capillary column (30 m × 0.25 mm, i.d. 0.25 μm film thickness)
Carrier gas	He (40 mL·min^−1^)
Source temperature	250°C
Oven conditions	ABA: 60°C (1 min) → 15°C·min^−1^ → 200°C (1 min) → 5°C·min^−1^ → 250°C (1 min) → 10°C·min^−1^ → 285°C (5 min)
	GA: 60°C (1 min) → 15°C·min^−1^ → 200°C (1 min) → 5°C·min^−1^ → 285°C (5 min)
	JA: 60°C (2 min) → 10°C·min^−1^ → 140°C (3 min) → 3°C·min^−1^ → 170°C → 15°C·min^−1^ → 285°C (8 min)
Injector temperature	200°C
Ionizing voltage	70 eV

#### Endogenous gibberellic acid (GA) analysis

The powdered whole plant samples (0.5 g) were used for extraction and quantification of GA_4_, GA_9_, GA_12_, GA_24_, and GA_34_ following an established protocol (Lee et al., 1998). Before column chromatography, deuterated GAs internal standards 20 ng of each (^2^H_2_GA_4_,^2^H_2_GA_9_, ^2^H_2_GA_12_, ^2^H_2_GA_24_, and ^2^H_2_GA_34_) were added to the samples. For quantification, the GC (Hewlett-Packard 6890, 5973 N mass selective detector) was equipped with HA-1 capillary column (30 m × 0.25 mm i.d. 0.25 μm film thickness), and oven temperature was programmed at 60°C for 1 min, then a rise of 15°C min^−1^ to 200°C, followed by rise of 5°C min^−1^ to 285°C (Table 2). Helium carrier gas was kept at a head pressure of 30 kPa. The GC was directly interfaced to a mass selective detector with an interface and source temperature of 280°C, an ionizing voltage of 70 eV, and a dwell time of 100 ms. Full scan mode (the first trial) and three major ions of the supplemented (^2^H_2_) GAs internal standards (the second trial), and the endogenous GAs were monitored simultaneously (standard GAs were purchased from Prof. Lewis N. Mander, Australian National University, Canberra, ACT, Australia). The endogenous GA_4_, GA_9_, GA_12_, GA_24_, and GA_34_ contents were calculated from the peak area ratios of 284/286, 298/300, 300/302, 314/316, and 506/508, respectively. The data were calculated in nano-grams per gram dry weight and the analysis was repeated three times.

### Endogenous salicylic acid (SA) analysis

The SA was extracted and quantified as previously described by Seskar et al. (1998). In brief, powdered whole plant samples about 0.2 g were sequentially extracted with 90 and 100% methanol by centrifuging at 10,000 × *g*. The combined methanol extracts were vacuum-dried. Dry pellets were resuspended in 2.5 ml of 5% trichloroacetic acid, and the supernatant was partitioned with ethyl acetate, cyclopentane, and isopropanol (ratio of 100:99:1, v/v). The top organic layer containing free SA was transferred to a 4 ml vial and dried with nitrogen gas. The dry SA was again suspended in 1 ml of 70% methanol. High performance liquid chromatography (HPLC) analyses were carried out on a Shimadzu with a fluorescence detector (Shimadzu RF-10AXL, excitation and emission, 305 and 365 nm, respectively) fitted with a C18 reverse phase HPLC column (HP hypersil ODS, particle size 5 μm, pore size 120 Å Waters). The flow rate was 1 ml/min (more detailed information revealed in Table 3).

**Table 3:** **HPLC operation conditions for the analysis of salicylic acid**.

**HPLC**					
Model	Shimadzu LC-10				
Detector	RF-10 Axl (fluorescence detector)				
Wavelength	Excitation 305 nm, emission 365 nm				
Column	HP hypersil ODS (particle size 5 μm, pore size 120 Å)				
Solvent A	100% MeOH				
Solvent B	100% water in 0.5% acetic acid				
	5 min	2.5 min	4.5 min	5 min	3 min
Gradient	A: 30%	A: 40%	A: 60%	A: 30%	A: 30%
	B: 70%	B: 60%	B: 40%	B: 70%	B: 70%
Flow rate	1 mL·min^−1^				

### Endogenous jasmonic acid (JA) analysis

The endogenous JA level was extracted and quantified according to a previously published protocol by Kim et al. (2014b). The same freeze-dried plant samples (0.1 g) already mentioned in the other hormone analysis methods were suspended in a solution of acetone and 50 mM citric acid (70:30 v/v) and [9, 10-^2^H_2_]-9, 10-dihydro-JA (25 ng) was added as an internal standard. The extracts were allowed to evaporate overnight at room temperature to avoid losses of volatile fatty acids. The resulting aqueous solution was then filtered and extracted three times, each time with 10 ml of diethyl ether. The pooled extracts were then loaded on a solid-phase extraction cartridge (500 mg of sorbent, aminopropyl). After loading, the cartridges were washed with 7.0 ml of trichloromethane and 2-propanol (2:1 v/v). The bound JA and the pertinent standard were eluted with 10 ml of diethyl ether and acetic acid (98:2 v/v). After evaporation of solvents and esterification of the residue with excess diazomethane, the sample was adjusted to 50 μl with dichloromethane. The extracts were then analyzed by GC/MS-SIM (6890 N network GC system and 5973 network mass selective detector; Agilent Technologies). Further detailed GC-MS information is mentioned in Table 2. To enhance the sensitivity of the method, spectra were recorded in the selected ion mode. For JA determination, the fragment ion was monitored at *m/z* 83 corresponding to the base peaks of JA and [9, 10-^2^H_2_]-9, 10-dihydro-JA. The amount of endogenous JA was calculated from the peak areas of endogenous JA compared to corresponding standards. Three replicates per treatment were used for the determination of JA.

### Determination of amino acid content

Freeze-dried whole plant samples (50 mg) were used for the determination of methionine and proline levels (Ishimoto et al., 2010). Free amino acid was extracted with 240 μL of 3% sulfosalicylic acid for 60 min followed by centrifugation at 12,000 × *g* for 10 min at room temperature. The obtained pellet was additionally extracted two times with the same amount of extraction solution at shaking for 60 min and were combined together and the resultant suspension were further processed for amino analysis. Samples of all the treatment were hydrolyzed in 5 ml of 6N HCl under vacuum in an ampulla tube for 24 h at 110°C. The suspension was then filtered and evaporated under vacuum. The solid residue was dissolved in 2 ml of deionized water and evaporated twice again. The final residue was dissolved in 10 ml of 0.01N HCl and then filtered with a 0.22-μm filter membrane and subjected to an automatic amino acid analyzer (L-8900 Hitachi, Japan). An amino acid standard mixture solution (type H) for automatic amino acid analysis was purchased from Wako Pure Chemical Industries Ltd. (Japan) and used for quantification of endogenous methionine and proline levels.

### Scanning electron microscope analysis

Thoroughly freeze-dried root samples were used for root anatomical inspection. The dry root samples were cut at 5 mm and sputter-coated with gold (JFC-110E ion sputtering device; EC&G, USA). Using the prepared samples, we obtained images from a field emission scanning electron microscope (FE-SEM, Hitachi S-4300, Japan).

### Lipid peroxidation

We determined lipid peroxidation to analyze the severity of damage in the roots cortex cells according to the method of Ohkawa et al. (1979). After 5th and 10th day of application of waterlogging stress, soybean roots were collected and 1 g of fresh sample was extracted with 10 mM phosphate buffer (pH 7.0). The mixture was heated in a water bath at 90°C for 60 min, then allowed to cool to room temperature. A 5-mL butanol/pyridine (15:1 v/v) solution was added and vortexed so that the mixture was separated into two layers. The upper organic layer was measured at 532 nm using a spectrophotometer. The level of lipid peroxidation was expressed as micromoles of MDA formed/mg of protein, and for quantitative analysis of MDA, tetramethoxypropane was used as an external standard.

### Statistical analysis

To evaluate waterlogging stress, all the data were statistically analyzed for standard error using Sigma Plot Software version 13.0. The mean values were compared using *T*-test at *P* < 0.05 and *P* < 0.01 (ANOVA SAS release 9.1; SAS, Gary, NC, USA).

## Results

### Plant growth characteristics data under waterlogging stress

To evaluate plant growth and developmental condition, we analyzed shoot length (SL), root length (RL), shoot width (SW), shoot fresh weight (SFW), and root fresh weight (RFW). In the 5 and 10 days after waterlogging treatment, the SL of WTL did not show a significant difference between the control and treatments, but SL of WSL was slightly reduced 10 days after treatment (DAT) in comparison with the control (Table 4). Shoot width (SW) showed the same tendency as the SL result, that is, only 10 DAT in WSL revealed a slight decrease (Table 4). Root length (RL) did not differ between the treatment and control, but SFW and RFW differ. SFW of WTL did not show any difference between control and treatment conditions 5 DAT; however, it was significantly decreased at 10 DAT. In the WSL, SFW was always lower after waterlogging stress than in the control plant (Table 4). Root fresh weight (RFW) was reduced in all waterlogging application comparisons with control plants (Table 4).

**Table 4:** **Effect of waterlogging stress on shoot length, root length, shoot width, and their respective weights of WTL and WSL**.

**AN^a^**	**DAT^d^**	**TM^e^**	**SL^h^(cm/p^i^)**	**RL^k^(cm/p)**	**SW^l^(cm/p)**	**SFW^m^(g/p)**	**RFW^n^(g/p)**
WTL^b^ (PI408105A)	5	C^f^	11.5±1.3 ns^j^	13.7±0.2 ns	4.5±0.2 ns	12.0±0.2 ns	5.3±2.1
		W^g^	11.2±0.7 ns	14.2±0.8 ns	4.7±0.8 ns	10.8±3.9 ns	3.2±1.6^*^
	10	C	17.0±1.7 ns	16.5±1.4 ns	5.3±0.5 ns	16.2±1.9	5.7±0.9
		W	16.8±0.1 ns	16.4±1.2 ns	5.1±1.0 ns	12.0±4.1^*^	4.7±1.6^*^
WSL^c^ (S99-2281)	5	C	17.8±2.7 ns	18.6±1.6 ns	3.7±0.2 ns	11.5±1.5	6.7±3.5
		W	19.3±0.7 ns	19.2±0.7 ns	3.8±0.2 ns	8.3±0.2^*^	3.1±0.9^*^
	10	C	25.6±3.0	21.1±1.1 ns	4.3±0.1	15.0±0.9	6.3±1.7
		W	22.8±3.2^*^	20.4±0.9 ns	4.8±0.7^*^	13.4±2.5^*^	5.6±1.5^*^

*Each data showed mean ± standard deviation (n = 20) and data was collected by three replication*.

* *shows the values are significantly different (P < 0.05)*.

a *Accession number*;

b *Waterlogging tolerance variety*;

c *Waterlogging susceptible variety*;

d *Days after treatment*;

e *Treatment*;

f *Control*;

g *Waterlogging*;

h *Shoot length*;

i *Plant*;

j *No significant difference*;

k *Root length*;

l *Stem width*;

m *Shoot fresh weight*;

n *Root fresh weight*;

According to Shimamura et al. (2014), adventitious roots develop well when plants faced water stress condition. For this reason, we surveyed adventitious roots, with the data shown in Figure 1A. Adventitious roots were well developed in both cultivars as time went on, but it was better developed in WTL than in WSL (Figures 1B–E).

**Figure 1:**
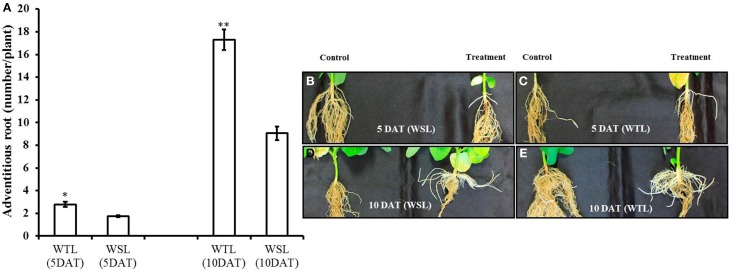
**Number of adventitious root and images of adventitious roots at 5 and 10 days after waterlogging stress**. In the figure, **(A)** indicates the number of adventitious roots and WTL indicates the waterlogging tolerant line (PI408105A), and WSL indicates the waterlogging susceptible line (S99-2281). **(B–E)** Indicate the images of adventitious roots and in each picture, the control plant is on the left, and waterlogging treated plant on the right. In **(A)**, star marks indicate a significant difference between control and treatment at *P* < 0.05 or *P* < 0.01. Data show average ± standard error (*n* = 20) and was collected over three replications.

### Endogenous ethylene production and methionine contents

**Figure 2:**
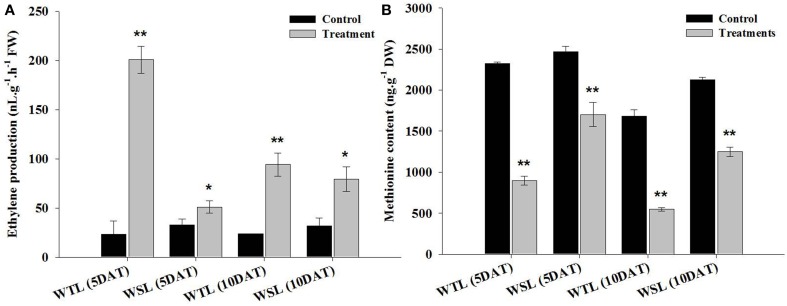
**Ethylene production (A) and amino acid content (B) in two soybean lines (WTL and WSL) after 5 and 10 days of waterlogging treatment**. In the figure, WTL denotes the waterlogging tolerant line (PI408105A) and WSL denotes the waterlogging susceptible line (S99-2281). Data is the average ± standard error (*n* = 3). The ^*^ and ^**^ shows the values are significantly different using P < 0.05 and P < 0.01 respectively.

Endogenous ethylene is known to be a key hormone involved in stress response. Particularly, it is a main regulator in waterlogging and submergence stresses because it triggers a response of hormones regulation, enzyme activity, and gene expression. The ethylene production under waterlogging stress is shown in Figure 2A. At 5 DAT, ethylene production was significantly increased in WTL (8.6-fold increase) in comparison with WSL (1.6-fold), and this tendency was observed at 10 DAT. Ethylene production increased 4-fold in WTL after 10 days of treatment, whereas it increased 2.5-fold in WSL (Figure 2A).

Methionine is an amino acid, known to be a precursor of ethylene. For this reason, we analyzed methionine content and showed the results in Figure 2B. After waterlogging stress, ethylene production was greatly increased, whereas methionine content significantly reduced after stress. The methionine content was approximately reduced by 61.4% in WTL at 5 DAT, but during the same stress treatment, methionine content was decreased by 31.1% in WSL. Thus, the range of methionine decline was higher in WTL than WSL. At 10 DAT, methionine contents was 67.6 and 41.3% decrease in WTL and WSL, respectively (Figure 2B).

Therefore, ethylene production was significantly increased after waterlogging stress, and especially increased in WTL as opposed to WSL during 5 and 10 days after waterlogging treatments. On the other hands, methionine content was more reduced after waterlogging application than in the control. Especially, the range of reduction of methionine contents was higher in WTL than in WSL in all waterlogging treatment periods (Figure 2B).

### Endogenous abscisic acid (ABA) levels and proline contents

**Figure 3:**
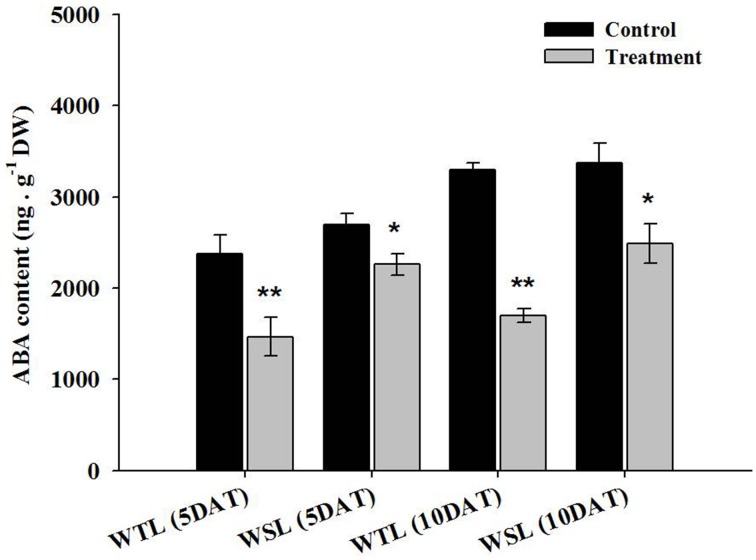
**Endogenous ABA content in the two soybean lines after waterlogging treatment**. In the figures, WTL denotes the waterlogging tolerant line (PI408105A) and WSL indicates the waterlogging susceptible line (S99-2281). In the figure, star marks indicate a significant difference between control and treatment at *P* < 0.05 or *P* < 0.01. Data is the average ± standard error (*n* = 3).

The main role of ABA in plants is the mediation of stomata by regulation of the size of guard cells, so water potential in the plant can be regulated. For this reason, ABA is known as the key hormone in water stress response. In addition, according to recent research, endogenous ABA is involved in aerenchyma cell development in roots under water stress (Shimamura et al., 2014). Thus, we analyzed endogenous ABA content after the application of waterlogging stress, and the results are shown in Figure 3.

At 5 and 10 DAT, all ABA levels decreased. As specifically mentioned, ABA level in WTL was approximately 38.1 and 48.5% decreased at 5 and 10 DAT, respectively, whereas ABA level in WSL was decreased by 16.3 and 26.2% at 5 and 10 DAT, respectively. Therefore, reduction of ABA content between WTL and WSL showed that ABA was more decreased in WTL than WSL under all waterlogging treatments (Figure 3). The proline was known as a regulator in various stress conditions such as high and low temperature, UV stress, salt stress, drought stress, and water stress (Szabados and Savouré, 2010). According to Szabados and Savouré (2010), if plants are faced with stress conditions, proline accumulates in plant cells through various biosynthetic and catabolic pathways, and thus, plants enhance their stress resistance.

**Figure 4:**
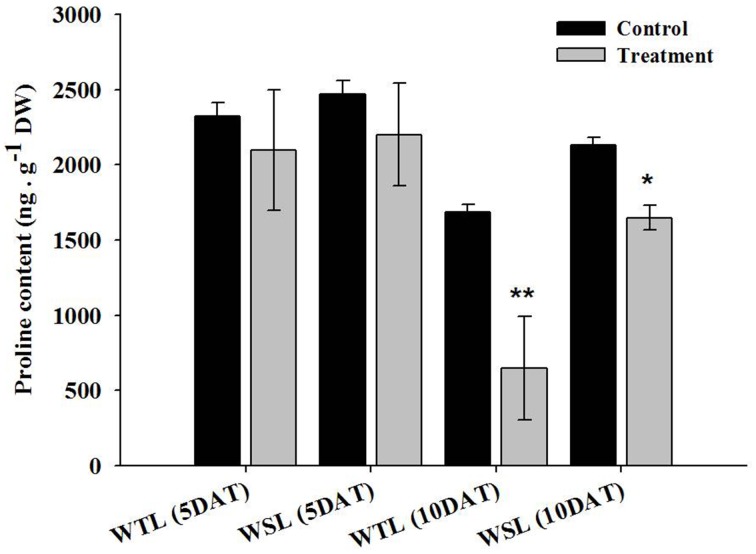
**Proline contents in the two soybean lines after waterlogging treatment**. In the figures, WTL denotes the waterlogging tolerant line (PI408105A) and WSL indicates the waterlogging susceptible line (S99-2281). In the figure, star marks indicate a significant difference between control and treatment at *P* < 0.05 or *P* < 0.01. Data is the average ± standard error (*n* = 3).

The proline content after waterlogging stress is shown in Figure 4. At 5 DAT, proline contents were not significantly different, but at 10 DAT, proline contents showed a difference between WTL and WSL. The proline content was decreased in both lines after 10-day waterlogging treatment, and most significantly reduced in WTL, whereas proline was slightly decreased in WSL (Figure 4).

### Endogenous gibberellic acid (GA) levels

**Figure 5:**
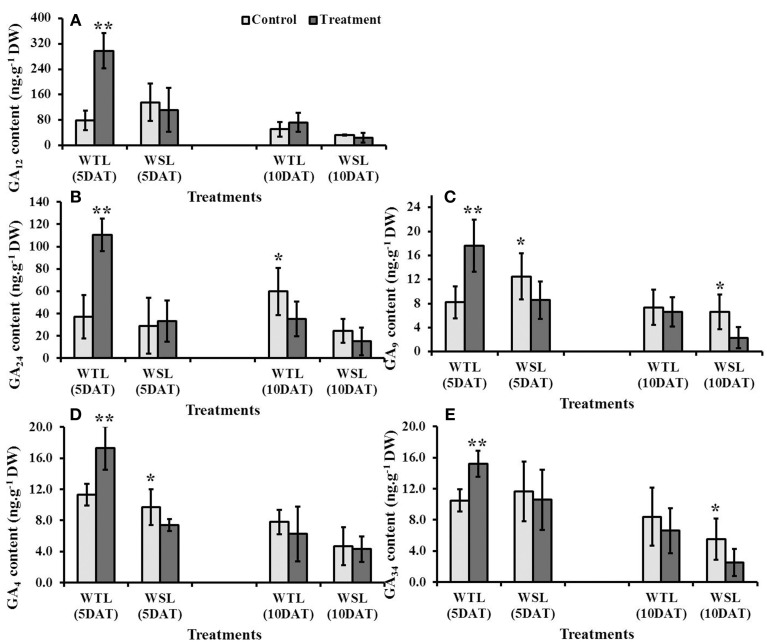
**Endogenous gibberellic acid (GA) contents in two soybean lines after waterlogging treatment**. The regulation of different gibberellic acids is shown as **(A)** G_12_, **(B)** G_24_, **(C)** GA_9_, **(D)** GA_4_ and **(E)** GA_34_. In the figures, WTL indicates the waterlogging tolerant line (PI408105A) and WSL revealed waterlogging susceptible line (S99-2281). In the figure, star marks indicated significantly difference between control and treatment at *P* < 0.05 or *P* < 0.01. Data is the average ± standard error (*n* = 3).

Of the plant hormones, GA especially can regulate various physiological responses, such as regulation of plant growth and development, induction of seed germination, control of flowering, and tolerance to stress conditions. Analyzed GA content was significantly high in WTL at 5 DAT, whereas mostly it was not significantly high at 10 DAT after waterlogging (Figure 5). In contrast, GA in WSL decreased after the waterlogging treatment (Figures 5B–E).

### Endogenous jasmonic acid (JA) and salicylic acid (SA) levels

**Figure 6:**
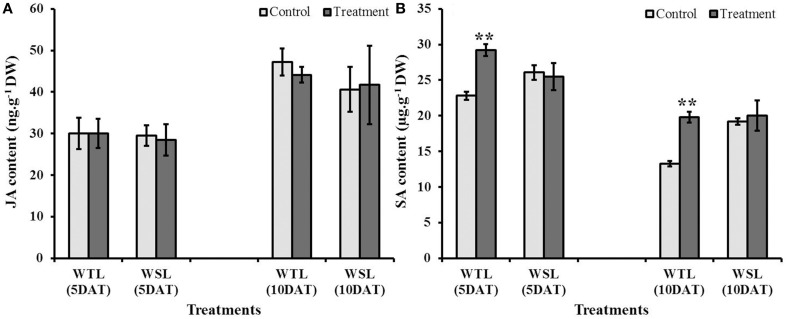
**Endogenous jasmonic acid (JA) (A) and salicylic acid (SA) (B) contents in two soybean lines after waterlogging treatment**. In the figures, WTL indicates the waterlogging tolerant line (PI408105A) and WSL denotes the waterlogging susceptible line (S99-2281). In the figure, star marks indicate a significant difference between control and treatment at *P* < 0.05 or *P* < 0.01. Data is the average ± standard error (*n* = 3).

Endogenous JA and SA are stress response hormones, so both hormones are accumulated under abiotic and biotic stress conditions. Further, these hormones have been known to be highly linked with ethylene production, and thus, plants enhance their stress resistance through interactions among hormones (ethylene, JA, and SA). Endogenous JA and SA contents after stress conditions are shown in Figure 6. JA content at 5 and 10 DAT was not significantly different between the control and treatment in both soybean lines (Figure 6A). On the other hand, SA level in WTL showed a significant difference from the control. Under waterlogging stress conditions, SA contents at 5 and 10 DAT in WTL were significantly higher than that under non-waterlogging condition, whereas the SA levels were not different between waterlogged and non-stress conditions in WSL (Figure 6B).

### Root anatomical inspection

**Figure 7:**
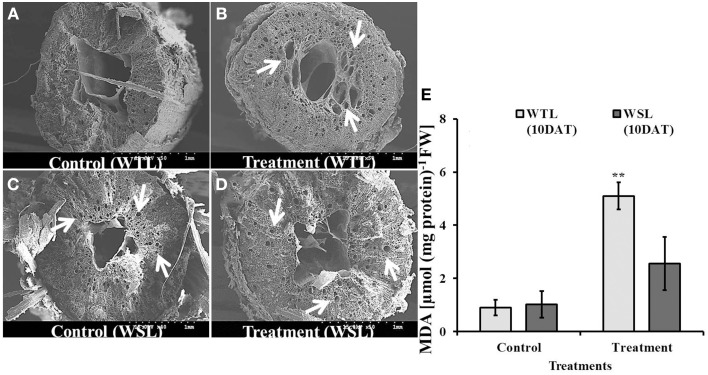
**The images of root section (A–D) and MDA (E) in root after 10 days waterlogging treatments**. In the figure, WTL indicates the waterlogging tolerant line (PI408105A) and WSL indicates the waterlogging susceptible line (S99-2281). The arrows in **(B–D)** indicate aerenchyma cells in the root. **(E)** Shows lipid peroxidation (MDA) in the root and star marks indicate a significantly difference between control and treatment at *P* < 0.05 or *P* < 0.01. Data is the average ± standard error (*n* = 3).

In waterlogged and submergence conditions, soil pore space was covered with water so plants faced hypoxia. Under normal conditions, plants can uptake oxygen from the soil through the root, which is used in energy generative by the process of respiration. However, under flooding conditions, plants cannot take up oxygen from soil. In order to facilitate oxygen uptake, the constitution of plant root cells changes to adapt stress conditions. For these reasons, we inspected the root cells of soybean plants after 10 days of waterlogging treatment. As shown in Figure 7, aerenchyma cells were observed in WTL compared to WSL, and the number of aerenchyma cells and size of aerenchyma cells were higher in the waterlogging treatment than the control at WTL (Figures 7A,B), while development of aerenchyma cell was not different between the control and treatment in WSL (Figures 7C,D). Therefore, aerenchyma cells were better developed in WTL than in WSL. To support the difference in development of aerenchyma cells, we analyzed lipid peroxidation (MDA) (Figure 7E) and no change was found between WTL and WSL without waterlogging treatment. Nonetheless, WTL showed greater levels of lipid peroxidation than the WSL under waterlogging stress.

## Discussion

Water logging stress in soybean mainly restricts seed productivity and so several waterlogging tolerant varieties have been developed (Reyna et al., 2003; Cornelious et al., 2005). In order to understand waterlogging tolerance mechanism and to identify QTLs in flooding tolerance, a few RILs were developed, which have been subsequently used in identification of QTLs (VanToai et al., 2001). Though, some QTLs were identified, but our findings are not in agreement with those of previous studies (Reyna et al., 2003). Flooding stress in soybean plants and the environmental factors including temperature and humidity during stress period, and disease induced by *Phytophthora* species were linked with the induction of stress tolerance responses (Reyna et al., 2003; Cornelious et al., 2005; Nguyen et al., 2012). In spite of various research works, still the QTLs for using marker-assisted selection (MAS) had not been identified. Therefore, the current experiments were conducted to contribute toward the understandings of waterlogging stress tolerance and might help in the discovery of QTLs to use in MAS.

Current investigation was conducted on two distinct soybean waterlogging tolerant and waterlogging susceptible lines. After 5 and 10 days of waterlogging stress application, plants of both soybean lines showed differences between the control and treatment groups. The fresh and dry weights of shoots and roots in both soybean lines were considerably decreased than that of non-waterlogging treated plants; however, the reduction rate was higher in WSL than in WTL. Our results validates that WSL (S99-2281) is more sensitive to waterlogging stress as previously reported by Nguyen et al. (2012) and justifies the use of soybean varieties with contrast characteristics in order to understand the tolerance through comparisons of physiological metabolism.

Botanically, a root is the underground organ of plant with primary function of absorbing minerals and moisture from the soil, as well as to provide firm anchorage (Bacanamwo and Purcell, 1999). In contrast, the adventitious root develops or grows in an unusual location, like a stem or leaf-derived cell. An adventitious root formation normally occurs in monocotyledons; however in other plant species, these are developed to adapt or survive in response to stress conditions (Li et al., 2007; Yang et al., 2013). In this study, adventitious roots in both soybean lines started to develop after 5 days of waterlogging treatment; however, significant difference were revealed in number of adventitious roots between WTL and WSL after 10 DAT. The adventitious roots must be well developed under stress conditions such as drought and flooding in order to survive, and this internal process is induced by signal transcription factors. Under waterlogging stress condition, the typical signal transcription is known to be triggered by all key plant hormones, particularly ethylene, auxin, and CK, involved in the development of adventitious roots (Werner et al., 2003; De Klerk and Hanecakova, 2008; Yang et al., 2013). Moreover, other biochemical products such as polyamines, peroxidase (POD), hydrogen peroxide (H_2_O_2_), calcium ion (Ca^2+^), nitric oxide (NO), and mitogen-activated protein kinase (MAPKs) were reported to regulate the development and growth of an adventitious root (Pagnussat et al., 2003; Lanteri et al., 2008; Yang et al., 2013). Therefore, our results may explain the difference in physiological responses involved in tolerant and susceptible lines regarding the development of adventitious roots. To further investigate these phenomena, measurement of ethylene evolution from whole plant and its precursor methionine content were focused. The findings of current results indicate that ethylene production was significantly higher in WTL than in WSL. An ethylene induces various plant physiological responses and is a key regulator of leaf senescence and fruit ripening process (Cuo and Ecker, 2004; Song et al., 2014). In addition, it can regulate other abiotic stresses and triggered an internal response at genetic level during flooding stress (Bailey-Serres et al., 2012). According to Visser et al. (1996), adventitious roots are induced by flooding conditions to channelize the transportation of air diffused in stem portion to the root. It was reported that under flooding conditions, auxin and ethylene triggered the development of adventitious roots. Visser et al. (1996) have witnessed that probably ethylene is a key regulator for the induction of adventitious root formation during flooding conditions as assumed by increased adventitious roots formation in *Rumex palustris* in response to ethylene application under flooding stress. Current results show that WTL produced more ethylene in waterlogged condition as compared to the production by WSL and non-waterlogging treated plants. Furthermore, low level of methionine in WTL shows that it may have been efficiently used for different physiological process. The assumption may be further strengthen as methionine through SAM (S-adenosyl-L-methionine) enhance cell division and synthesis of cell wall, chlorophyll, membrane, propylamino group (polyamines, spermidine, spermine), and glucosinolates (Roje, 2006; Kuznetsov and Shevyakova, 2007). Propylamino group plays an important role in proliferation, differentiation, and homeostasis of cells (Kuznetsov and Shevyakova, 2007). These results for ethylene production can possibly suggest one of the reasons for difference in significant development of adventitious roots between WTL and WSL.

To adapt to flood conditions, plant roots bring changes in internal anatomical structures. Under anoxic or hypoxic soil conditions, plant root cell walls are degraded by enzymes such as cellulase so that aerenchyma cells are developed. The formation of aerenchyma cells under flooding conditions can be considered a tolerance mechanism for long-term survival (Visser et al., 1996; Shimamura et al., 2014). As mentioned earlier, oxygen is very important for the generation of mitochondrial ATP production, and therefore, wetland plant species have well-formed aerenchyma cells in the root tip to facilitate the transport and intake of atmospheric oxygen (Shimamura et al., 2014).

Besides, ABA induces many physiological functions like promotion of suberin deposition in cell walls; it is a known regulator of drought and salt stress responses (Kim et al., 2014a,b; Shimamura et al., 2014). To survive flooding conditions, soybean plants need morphological changes in their roots, and in response, the aerenchyma cells are formed. However, for aerenchyma cells development, a root cell must be unsuberized, and therefore, the biosynthesis of suberin must be suppressed by the down regulation of ABA in soybean. Similar results were found in current experiments and the aerenchyma cells were well-developed in WTL roots with significant decrease in whole plant ABA contents compared to WSL and control. This down regulation of ABA after flooding treatment might suggest some explanation for better development of aerenchyma cells in WTL than in WSL.

In another context, development of aerenchyma cells could be defined as a form of programmed cell death (PCD) of cortex cells by internal cellular metabolism. Drew et al. (2000) argued that increase in ACC synthase and ACC oxidase are rapidly activated in maize root under hypoxia and consequently ethylene production is increased to trigger other signaling cascades and thus inducing plant cell death. According to Grichko and Glick (2001), soil flooding conditions inhibit the transportation of ACC synthase from root to shoot, and hence, the level is raised in root. Consequently, the lipid peroxidation is increased and thereby causes the destruction of the cell wall. The current experimental results revealed that WTL showed greater waterlogging tolerance than WSL, and this can be correlated with the difference in secondary metabolites synthesis such as endogenous hormones. Particularly endogenous ethylene production was significantly higher in WTL than in WSL, and therefore, it was hypothesized that ethylene may have acted on two physiological response for inducing waterlogging tolerance. This could be the initiation for the development of adventitious roots and the increased lipid peroxidation (MDA), as shown in Figure 7E and can be correlated with the growth of aerenchyma cells in roots.

Salicylic acid (SA) is well-known plant stress hormone, which induces systemic acquired resistance (SAR) or hypersensitive response (HR) during fungal or insect attack in addition of regulating various physiological responses such as flowering, thermogenesis (Yang et al., 2004), ion absorption (Zhang et al., 2003), and PCD (Brodersen et al., 2005). Plants under fungal or insect attack rapidly increased the SA content to enhance SA-induced PCD surrounding the attacked cell in order to reduce damage to other cells. Recently, Yang et al. (2013) has investigated that proper concentration of SA significantly increases the adventitious root formation. Current experimental results also showed the same trend, and SA contents were highly increased in WTL, 5 and 10 days after waterlogging treatments, and this increase in SA level could be one of the tolerance factors to waterlogging stress. It can be assumed that SA might have also regulated two different physiological responses. First, increased SA-triggered PCD responses in the root, and as a result, lipid peroxidation was increased in the root cell wall and thereby aerenchyma cells become developed in the root. Well-developed aerenchyma cells improve oxygen supply and may be a cause of inducing waterlogging tolerance. Second, accumulated SA may stimulate adventitious root primordium formation to further enhance resistance against waterlogging stress by inducing the development of numerous adventitious roots.

Gibberellins (GAs) are related with most of the plant's physiological responses, and its main role is the regulation of growth and development through the control of cell size and number (Kim et al., 2011). Gibberellins role has been authenticated as a key hormone for enhancing tolerance in rice plants and *Rumex palustris* under submerged condition. Ethylene response factor (ERF) is up-regulated under submergence conditions, and as a result, the GA level is up-regulated thereby causes the elongation of internode to bring out rice leaves from water surface for normal respiration (Voesenek et al., 2003; Xu et al., 2006; Fukao et al., 2011; Bailey-Serres et al., 2012). Likewise, current results confirmed these findings as the GA level was increased after waterlogging stress in our experiments. However, it cannot be inferred that how GA contributed to waterlogging tolerance, because this study focused on changes in endogenous hormones along the waterlogging stress time course and conducted hormonal analysis at 5 and 10 days after treatment. Additional research work is needed for in depth identification of GA roles and tracing the molecular events from survival to death under waterlogging conditions in soybean.

**Figure 8:**
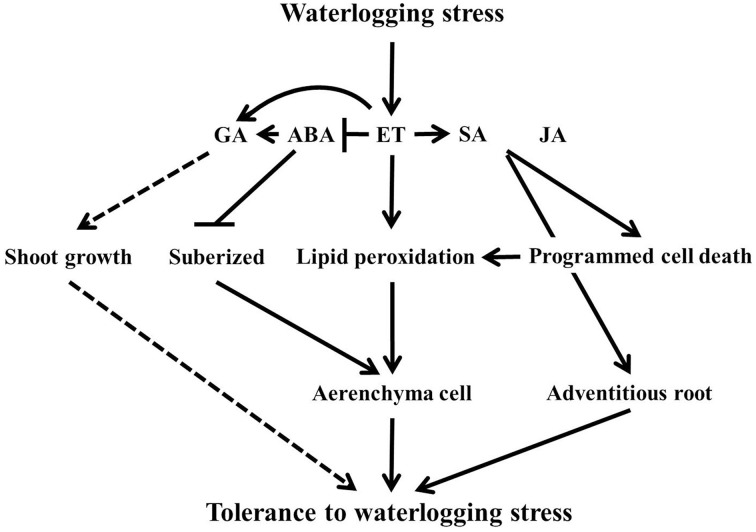
**The scheme of the waterlogging tolerance mechanism under plant hormones level**. In the figure, solid lines indicate induction or increase and dashed line arrows indicate putative response. The line with bars shows suppressed or inhibited.

It is concluded that flooding severely affect normal functioning of plants. To withstand this abiotic stress, plants bring in physiological changes for adaptation as shown by the hormonal and amino acid regulation, better development of aerenchyma cells and adventitious roots, and lipid peroxidation for waterlogging tolerance regulated by complex genetic mechanism (Figure 8). However, the difference in genetic background of tolerance and susceptible lines vary this complex genetic mechanism, which may contribute to the level of tolerance mechanism. It was revealed that flooding tolerance or masking the effect of flooding could be associated with quantitative trait loci (QTL). For instance, Nguyen et al. (2012) determined that resistance to injury or yield loss due to flooding or water logging tolerance in RIL may be genetically controlled and confer by the presence of genes located on four chromosomes. Furthermore, these two soybean lines were used as material for root-related transcription factors associated with flooding tolerance (Valliyodan et al., 2014). According to Valliyodan et al. (2014), they identified some different gene expression patterns involved in ethylene biosynthesis pathway. Therefore, further investigation is recommended at molecular level to dig out the genetic components and expression profile on suggested QTL enriched-chromosomes that associated with waterlogging stress tolerance particularly through hormonal modulation to maximize the level of tolerance for future breeding of crops. This will also reveal that many QTLs or hormonal level differences contribute to waterlogging tolerance.

## Author contributions

YK wrote the manuscript and also analyzed ethylene production; SH performed all the data collection such as plant growth characteristics, hormonal level, and amino acid contents; MW and AK supported the study by revising the manuscript; JHL performed inspection of root cell by using FE-SEM; JDL donated plant material; HN contributed to the development of the soybean variety; IL inspected all the experimental design and revised the manuscript.

### Conflict of interest statement

The authors declare that the research was conducted in the absence of any commercial or financial relationships that could be construed as a potential conflict of interest.

## Abbreviations

ABA: abscisic acid
GA: gibberellic acid
WSL: waterlogging susceptible line
WTL: waterlogging tolerant line.
